# Generativity as a heuristic for impact-driven scholars addressing grand challenges

**DOI:** 10.1177/14761270241239137

**Published:** 2024-04-03

**Authors:** Christopher Luederitz, Dror Etzion

**Affiliations:** McGill University, Canada; The University of Vermont, USA

**Keywords:** climate change, collaboration, organizational change, research and development, small business, sustainability, topics and perspectives

## Abstract

In this contribution, we theorize generativity as a heuristic for impact-driven management scholars seeking to address grand challenges through research. We use generativity to connote the engagement of diverse actors in pluralistic inquiry to create conditions for future flourishing. Our theorization applies a pragmatist worldview and builds on insights from the multidisciplinary literature on generativity to envisage researchers as agents of care, collective learning, and transformative change. We synthesize four tenets for researchers seeking both academic and real-world impact. These tenets can support researchers addressing grand challenges by guiding their efforts to diversify inputs, distribute agency, conduct experiments, and pursue prospective impacts. We illustrate generativity in action by drawing on our experience in a transdisciplinary research project on small- and medium-sized enterprises taking climate action in Canada. We show how the four tenets foster generativity to promote an inclusive understanding of grand challenges and a bias toward action, thereby providing an optimistic stance toward addressing issues of social concern.

## Introduction

Grand challenges such as climate change, resource depletion, poverty, and human rights pose new challenges to management scholarship and its aspiration to provide useful insights to society (George et al., 2016; Williams and Whiteman, 2021). A particularly acute challenge for researchers focusing on grand challenges is that, almost by definition, they aim to have an impact both inside and outside the halls of academia. Attaining this dual impact bedevils academic work on grand challenges, which are characterized by an ill-defined problem context, complexity, long-term implications, mutating stakeholder constellations, and contestation (Burns, 2014; Etzion et al., 2017). Consequently, to researchers, they often seem almost as difficult to study as they are to resolve.

Against this backdrop, some scholars have turned to generativity to produce new conceptualizations (Roulet and Bothello, 2021) theorizing (Langley, 2021) and tools (Pavez et al., 2021) for advancing impact-driven research on grand challenges. Others have suggested that new generative elements (Gatzweiler et al., 2022) and generative experimentation (Gehman et al., 2022) could change the way in which grand challenges are addressed. Here, we build on these studies to provide more practical guidance for researchers actively engaging with grand challenges. In the context of impact-driven research, we conceive generativity as an approach for engaging diverse actors in pluralistic inquiry oriented toward creating conditions for future flourishing. We argue that generativity can guide impact-driven researchers because it focuses on how individuals can act meaningfully in complex situations to create conditions for transformative change.

In this article, we seek to illustrate how researchers working on unclear and intractable challenges can draw on generativity to more effectively pursue impact-driven research. Importantly, and in contrast to much of the vast literature on participatory and engaged scholarship, we do not suggest a new methodology. Instead, our intent is to provide a practical approach for how researchers can imbue themselves and their research projects with generative aspirations and optimism when addressing issues of social concern.

We begin by reviewing the core characteristics that define grand challenges and distill their implications for researchers by building on the principles of philosophical pragmatism. This starting point positions the researcher as an individual who concurrently studies a problem and acts toward its mitigation. Next, we review the multidisciplinary literature on the concept of generativity to better understand how dedicated care, collective learning, and transformative change can flourish in complex situations characterized by fluidity and emergent opportunities. Synthesizing these insights into a heuristic, we develop tenets that encourage management scholars to gather diverse inputs and empower a broad variety of actors to jointly conduct concrete interventions that constitute the building blocks of transformative change. We illustrate the application of generativity in a large, transdisciplinary multi-year research partnership on small- and medium-sized enterprises (SMEs) and climate action in Canada. We conclude with reflections on the implications these insights hold for research impact commensurate with the enormity of grand challenges.

## Management in the context of grand challenges

As an “applied discipline,” management scholarship seeks to influence, modify, and assure organizational practices (Bartunek and Rynes, 2010: 110). In other words, to be impactful. This has been an elusive endeavor for decades, even when delimited to the world of organizations. The challenge is even more difficult when dealing with current, pressing problems. The impact of management research on grand challenges such as climate change is restricted because these problems extend beyond the local context and the influence of individual researchers. Critical questions, and self-examination, of how the results of academic studies may contribute to addressing grand challenges are often shunted aside in the hurly-burly of specific research projects and the publication of results. The literature on research impact does little to assuage these vexing concerns. It often focuses on theorizing and defining what counts as impact and struggles to escape tightly circumscribed interpretations. Overall, upon inspection, the literature on impact offers little guidance on how researchers should orient themselves to create conditions that support actors—and themselves—in addressing grand challenges.

In this section, we review core characteristics of grand challenges and the difficulties they create for impact-driven researchers. Next, we provide a brief overview of pragmatist philosophy, which forms the foundation of our approach to researcher impact. In line with pragmatism’s progenitors, we consider it a particularly potent approach for addressing issues of grave concern. With this baseline established, in the following sections, we introduce generativity as a heuristic for researchers to engage in a pragmatically grounded approach to tackling grand challenges, and thereby having an impact.

### Core characteristics of grand challenges

Increasingly, impact-driven scholars are confronted with the nature of grand challenges, characterized by effects that manifest across contexts, while inaction produces irreversibility of changed environments and exacerbates the magnitude of ramifications. Grand challenges present at least four obstacles for researchers seeking impact.

First, the framing of grand challenges is dynamic and might change over time, as new solutions are put forward or new stakeholders are involved (Stjerne et al., 2022). Over time different positions emerge on how to relate to and conceptualize the problem, and new opportunities arise for interventions. Accordingly, researchers working on grand challenges need to build on people’s diverging understanding and associated aspirations to explore the full range of solution options (Etzion et al., 2017). This means appreciating and incorporating the multiplicity of stakeholder goals as well as the possibility of contestation over the direction of change that accompany impact-driven research. Only if these dynamics are considered can truly transformative learning among stakeholders emerge, through the questioning of assumptions underlying diverging aspirations and the movement from individual-level understandings to collective actions (Armitage et al., 2008; Barth et al., 2023; Roulet and Bothello, 2021).

Second, because more than one pathway exists to address grand challenges, and roles and responsibilities are in flux throughout processes of problem-solving, it is unwise for decision-making to be centered on a single actor (DiVito et al., 2020; Quayle et al., 2019). Impact-driven researchers cannot limit the involvement of stakeholders to specific groups or time periods without the risk of undermining collective capacity to realize change. Consequently, the ability to take transformative action is necessarily distributed (Luederitz et al., 2021). Such emerging processes can only unfold if spaces for new actors and roles are created to enable collective efforts to evolve (Etzion et al., 2017).

Third, grand challenges are interconnected (Gümüsay et al., 2022) without a clear demarcation—spatially and temporally—of where they start and end. For example, climate change, biodiversity loss, and social inequalities are interconnected issues that are not bounded by specific geographies, cultures, or sectors. Any attempt to solve a given “problem” cannot proceed without recognizing and acting on this interconnectedness. Ignoring the multi-directional influence between systems ultimately impedes successful mitigation of an issue (Sachs et al., 2019). This creates a challenging situation for researchers because a local approach is needed to engage meaningfully in social processes, while at the same time moving beyond isolated understandings is a prerequisite to viewing multifaceted problems holistically.

Fourth, actors working on mitigating grand challenges create long-term and multifaceted change processes that, like the original “problem” itself, are ill-defined with unforeseen social implications (Rittel and Webber, 1973; van der Leeuw et al., 2012). Accordingly, researchers need to match these inter- and intragenerational implications with a future orientation beyond the singular focus on immediate results. With isolated solutions having little success in altering problematic constellations of grand challenges, impact-driven research must necessarily shift its focus from addressing symptoms to creating conditions supportive of change processes. Only with attention devoted to care and prospering relationships can a research practice evolve that indeed contributes to long-lasting, concerted efforts in support of sustainability (Caniglia et al., 2023).

Universities are organizations well equipped to surmount these daunting challenges. Described as “anchor organizations” within society, universities fulfill a unique role as boundary spanning, knowledge mobilizing, and expertise developing organizations (Birch et al., 2013: 7). They can and often do provide space and opportunity for researchers to partake in addressing grand challenges (Cash et al., 2003; Hart et al., 2015). At the same time, researchers often act as spectators and analysts of change, situated at some remove from the problems they study (Cunliffe and Pavlovich, 2022). But if researchers work solely in “the ivory tower,” they are limited in their ability to create conditions for change and mobilize practitioners across geographies, cultures, and scales to disrupt established conventional understandings and practices that hamper more sustainable ways of organizing (Carton et al., 2023; Trencher et al., 2017). A close engagement seems necessary (Etzion and Gehman, 2019). Yet, how can researchers act as agents of care, collective learning, and transformative change in the context of grand challenges? We turn to philosophical pragmatism to develop the conceptual foundation for understanding what characterizes impact-driven researchers.

### Insights from philosophical pragmatism for researchers addressing grand challenges

Philosophical pragmatism has gained increased attention in the context of grand challenges. Pragmatists’ reflexive nature, process-oriented inquiry, and learning-centered underpinnings have given rise to ameliorated interpretation and development of new approaches (Ferraro et al., 2015), methodologies (Kistruck and Slade Shantz, 2022), and theories (Simpson and den Hond, 2022). This scholarship takes an outward orientation. It venerates the academic endeavor and its outputs, but not the researcher or knowledge for knowledge’s sake.

By contrast, here, we expand the implications of philosophical pragmatism to impact-driven researchers themselves. In doing so, we follow an inward orientation to examine the implementation of pragmatism in research projects. Instead of developing a new research approach, our aim is to employ pragmatist insights to better understand the role researchers themselves can play and how we as scholars should orient ourselves toward grand challenges.

Of course, philosophical pragmatism has already established important insights for researchers aiming to create impact. In particular, pragmatism posits that knowing and acting are intertwined and therefore researchers are not spectators or translators but first and foremost problem solvers (Vo et al., 2012). Pragmatists understand impact-driven research as the outcome of a process of inquiry and problem-solving pursued through ongoing experimentation—where learning must be continuous and unceasing. In addition, researchers are not solitary actors in this endeavor but, as Peirce pointed out, advance “toward the truth as part of a community of inquirers trusting to the multitude and variety of our reasonings rather than to the strength of any one” (Choo, 2015: 53). Continuous learning thus enables researchers to collaborate with others when constructing and reconstructing the contextual realities in which actions take place, and improves their capacity to act through experimentation and deliberation (Ansell, 2011; Herrigel, 2010).

The dynamic understanding of learning as conceptualized by philosophical pragmatism highlights the agency that impact-driven researchers have over the direction of problem-solving which is “brought to consciousness and made a factor in determining present observation and choice of ways of acting” (Dewey, 1916: 106). Conceiving of agency in problem-solving processes in this way suggests that impact-driven researchers must be “vital beings who contribute very actively to the creation of the social world that defines them” (Herrigel, 2010: 19). However, due to the complexity and scale at which grand challenges evolve, the sphere of direct influence of researchers is naturally limited. We thus propose that the concept of generativity could complement pragmatists’ insights into problem-solving to understand how researchers can leverage transformational change in ways that unfold beyond the narrow confines of individual research projects.

## Generativity

Originating in psychology, generativity refers to personal development focused on caring for and guiding the next generation (Peterson et al., 1997). Erikson (1950) coined the term as he articulated a theory of lifespan development. He contrasted generativity with stagnation to explain a person’s efforts to nurture the next generation by imparting skills and mindsets capable of navigating future challenges. Erikson emphasized that this task involves recognizing the changing needs of future generations and equipping them to become generative persons themselves, which in turn requires thoughtful decisions about devoting time and energy to certain causes and rejecting others (Peterson et al., 1997). Accordingly, generativity is a personal-level trait with society-level implications (Warburton and Gooch, 2007). It is driven by a desire for community (i.e. “to relate to others in loving, caring, and intimate ways, even to be at one with others”) and agency (i.e. “to assert, expand, and develop the self in a powerful and independent way”; McAdams and de St. Aubin, 1992: 1005), which anchors the individual in time by connecting one’s past with the act of reciprocating and paying forward generosity and kindness (Hostetler, 2009). While Erikson initially theorized generativity as the seventh stage of personal development, later research showed it applies to all of adulthood (McAdams and de St. Aubin, 1992) and that it operates multi-directionally across ages and identities as people commit to and practice “learning, advocacy, and care” (Chazan and Baldwin, 2021: 77).

In parallel to research in psychology, contributions to education and teaching have examined the generative capacity of knowledge and the process of how creative actions are learned. For learning to be generative, it requires a person to continuously apply and add to one’s knowledge in order to be able to address new problem situations (Franke et al., 2001). This creativity to navigate changed contexts essentially establishes a new practice which emerges from “an orderly competition among previously established behaviors” as generative theory suggests (Epstein, 1999: 759). Building on these insights, research on education has conceptualized generativity as a process instead of an output or an end unto itself (Brito and Ball, 2020). For example, for educators to positively affect the lives of students, Ball’s (2009) model of generativity theorized that the combined application of insights gained from interactions with others, ongoing research, and situated experiences result in “a process of self-perpetuating change” (Ball, 2009: 48; Liu and Ball, 2019). This places particular emphasis on reflexivity, agency, and advocacy to build the capacity to continuously learn and problem-solve (Ball, 2009).

The emphasis on learning as a key characteristic of generativity has been further developed in organizational research and teaching. Organizational research theorized generativity against the backdrop of the “learning organization” to understand how an organization develops the capacity to reinvent itself and, with it, the context in which it is embedded (Senge, 1990: 1). Here, generativity constitutes an approach in which organizations frame and solve problems through continuous experimentation and evaluation (McGill et al., 1992). Whereas adaptability constitutes a response to external changes, aligning behaviors and adjusting outputs to match the new context, generativity can identify opportunities for effective action outside the organizational boundary (McGill et al., 1992; Senge, 1990).

Research on technology and digitalization has moved beyond the individual and organizational context in conceptualizing generativity to better understand network characteristics. Here, generativity describes “a technology’s overall capacity to produce unprompted change driven by large, varied, and uncoordinated audiences” (Zittrain, 2009: 198). Whereas other work on generativity emphasized focal actors—be it an individual caring for others or an organization reinventing itself—in the context of technology, it unfolds across network nodes. Put differently, generativity involves the metamorphosis of an ability or function into a new output “without any input from the originator” (Tilson et al., 2010: 750). It explicitly implies fostering the emergence of new system structures that are unknown and producing new functions even if this results in unintended consequences (Kretzer and Maedche, 2014: 210).

Aside from the discernment of generativity in different domains, scholars have also begun exploring its implication for research practice itself. Ball (2009) noted that scholars could themselves become generative. Researchers, she argues, have the privilege to combine their academic insights with their personal experiences and the local knowledge of case-based studies to improve a system under consideration. Ball (2012) further conceptualized generativity as a zone, in line with how it is applied in psychology (McAdams and de St. Aubin, 1992). “The concept of a zone allows individuals to enter in at different points—based on one’s ability, one’s commitment, one’s own level of advocacy, one’s level of current knowing” (Ball, 2012: 289). Accordingly, the generative researcher influences and inspires others’ knowing and doing.

Common to the diverse literature on generativity is the understanding that this concept centers on the notion of potentiality and opportunity and their unfolding in the future to shape conditions beneficial for change. Generativity takes place on two levels—that of the individual and the collective. Indeed, some scholars (e.g. Erikson, Franke, Ball) demonstrated how individuals can foster generativity, for example, by raising the next generation, through personal development, and by educating others. Yet, for this capacity to be generative it has to materialize at a broader, collective level. Generativity shapes the context under which actions are carried out. As illustrated (e.g. Senge, Zittrain, Ball), generativity creates subsequent and emergent effects, which open new spaces for action.

## A heuristic for generative management research

These epistemologies and theories of generativity have been ascendant in recent management research. In particular, Pavez et al. (2021)—building on Cooperrider’s (2021) idea of prospective theorizing—argued that research should focus on outlining a new kind of organizing, one that is different from what exists in the past or present. They thus called on management scholars to engage with what they call generative scholarship. Researchers following this approach would prioritize the “unleashing of current knowledge and ideas, grounded research, and theoretical imagination to co-envision, co-open, and co-create new and better possibilities to enliven human organizations—within their interconnected eco-systems—and building a flourishing world” (Langley, 2021; Pavez et al., 2021: 462; Roulet and Bothello, 2021). While such aspirations are certainly compelling, they do not provide much guidance for researchers grappling with acting and orienting themselves in research projects addressing grand challenges.

Our position is that generative research is an approach for engaging diverse actors in pluralistic inquiry oriented toward creating conditions for future flourishing. To unpack this definition and offer a heuristic for management scholarship, we synthesize, from the literature review in the previous section, core tenets that we believe enable generativity and yield impactful research. We develop four tenets with the intent to guide researchers in gathering diverse inputs and empowering a broad variety of actors to jointly conduct concrete interventions that constitute the building blocks of transformative change.

The first tenet centers on *diversifying inputs*. We specify this tenet by bringing together insights from teaching and technology on how generativity emerges from the colliding and merging of diverse influences to ensure continuous learning. On a personal level, generativity is the outcome of personal interactions, new insights, and situated experiences, while on a collective level, it results from a large number of uncoordinated interactions fulfilling diverging intentions.

The second tenet focuses on *distributing agency*. For defining this tenet, we combine insights from research in psychology and technology on how generativity stimulates sharing of agency. In psychology, generativity defines the ability of individuals to instill in others the capacity to take self-directed actions. Generativity in the context of technology describes network dynamics enabling interconnected groups to pursue actions beyond those that determined their initial formation.

The third tenet guides researchers on *conducting experiments*. We draw on insights from organizational learning which suggests that a generative mode of operation is one that seeks to transform a given context through experimentation. Experimentation is key to generativity because it enables refining understandings and intentions by means of intervention, transforming the environment in which action and learning are embedded.

The fourth tenet focuses on *pursuing prospective impacts*. All streams of research on generativity highlight the need for increasing the potential and opportunity for beneficial conditions to unfold in the future. For example, generativity unfolds as individuals pay forward benefits to empower future generations and it leads to the emergence of new structures outside of the realm of control of originating actors.

In brief, our argument is that adopting these four tenets of generativity as an organizing approach builds a heuristic for researchers to foreground the complex, ambiguous, and challenging realities of change processes and enable transformative action on grand challenges. Importantly, the four tenets do not follow a specific order but reinforce each other and are equally important to all research activities as we demonstrate in the case description of the PIVOT project.

### Tenet 1 on leveraging the local setting: diversify inputs

Generativity encourages continuous updating of goals and aspirations of research projects by considering multiple inputs. This tenet calls upon generative researchers to gather diverse insights and stimuli when orienting research toward transformative change. At the level of researchers, these inputs can encompass personal experiences, interactions, and reflections (Ball, 2009; Liu and Ball, 2019). For the broader research project, stimuli can emerge from changing properties, multi-directional influences of events, and the uncoordinated actions of people engaging with each other (Tilson et al., 2010). People are linked and interact through nonlinear and unpredictable interrelationships, which are shaped by how people perceive themselves and others (Langley and Tsoukas, 2012; Mead, 1932). The goals and aspirations that emerge and grow from these interactions drive the innovation, creativity, and emergence associated with generativity (see, for example, Ball, 2009; Epstein, 1999).

This approach differs substantially from many other impact-driven efforts, which often expend significant effort on working toward unifying goals. Projects in this vein are typically developed around a “shared” problem understanding to define common objectives which stakeholders are expected to pursue in collaboration. Yet, the complexity of grand challenges undermines this teleological theorization of actions, because the uncertainty surrounding impact-driven research projects makes calculated strategizing impossible (Joas, 1992). The problem state and the desired solution of grand challenges are never fully known and attempts to tame this uncertainty through research projects will ultimately fail. Generativity suggests a different vantage point to deal with highly complex and uncertain problem situations. Instead of reducing possible strategies and solution options by finding a common denominator among stakeholders, generativity encourages researchers to pursue multiple—complementary or contradicting—opportunities when addressing grand challenges. Indeed, people’s motivations to engage in research projects are varied and their “true” aspirations cannot be known in advance.

When seeking generativity researchers must therefore neither assert nor contest competing viewpoints. Generativity avoids placing diverging impact pathways at odds. Rather, it seeks to find opportunities for combinatory effects. For example, conversations over transitions to sustainability might diverge when some advocates argue for ecological modernization and the green economy, whereas others favor economic degrowth and decentralized initiatives. At the societal level, these pathways appear at first glance to be in conflict, but they can also be understood as pursuing different intervention points and thereby being complementary (Luederitz et al., 2017a). A strength of the green economy pathways is harmonizing institutional fragmentation and offering entry points for application at higher organizational levels. Decentralized initiatives can succeed in addressing localized issues, such as transforming regional energy systems and encouraging citizens to experiment with new practices. Accordingly, the role of researchers is less the identification of the “right” transition pathway, but rather the ability to remain fluid and provide spaces where shared interest can materialize (Stjerne et al., 2022). Embracing differences in this way can energize initiatives that otherwise are at risk of marginalizing contrary pathways and excluding alternative voices (Leipold, 2021).

Implementing this tenet in projects means researchers embrace openness toward differences and pluralistic understanding of change processes. Diversifying inputs requires researchers to engage different ways of knowing, and to incorporate perspectives and interactions different from one’s own when developing and carrying out projects. This includes reflexivity toward values, how they are articulated and whose values are considered or excluded from research.

### Tenet 2 on activating actors: distribute agency

Generativity suggests broadening the locus of control over the research to build a support system beyond a small number of central actors (Zittrain, 2009). This tenet calls upon generative researchers to decentralize ownership of research projects to encourage individuals and collectives to become empowered actors. Distributing agency can be a useful risk mitigation strategy because research projects addressing social concerns are often characterized by uneven organizational capacity, both among practitioners, and also between practitioners and researchers (Breu and Hemingway, 2005; Harrison, 2011; Smith et al., 2010). Notwithstanding the risks of cooptation and “losing control,” enabling as many other actors as possible to do as much as they can or want is generally a good starting point for work on grand challenges.

Importantly, distributed agency goes beyond the direct involvement of key practitioners in research projects and focuses attention on building a system that enables continuous engagement of a broad set of actors—even if relationships are adversarial—according to their abilities and constraints (Luederitz et al., 2021; Roulet and Bothello, 2021; Sharma and Bansal, 2020). For researchers to distribute agency across and beyond the immediate project boundaries necessitates involving actors that may not be “officially” associated with the particular issue, or that may not have explicitly articulated their interest. This contrasts with existing impact-driven approaches that often seek to separate the space practitioners and researchers inhabit and centralize control with the latter, who act as initiators of projects and hence maintain oversight of processes and outputs (Grant et al., 2008; Learmonth and Harding, 2006; Smith et al., 2010). These dynamics risk portraying researchers as the key actor in solving problems, a framing not necessarily shared by practitioners (Varkarolis and King, 2017).

Guided by generativity, researchers can sunder this division of labor by embracing the evolving nature of research projects and the changing responsibilities over time accordingly. Actions on grand challenges are not confined to specific activities (such as political advocacy or consumer choices) but span all forms of private, public, and civil organizing (De Meyer et al., 2021). The process of addressing grand challenges—including identification of inadequate responses, new problem formation, and revised mitigation attempts—requires multi-directional relationships between diverse organizations with different purposes and resources (Kaufmann and Danner-Schröder, 2022). Building on these considerations, people’s involvement in research projects is necessarily dynamic, ranging from information and consultation, to collaboration and empowerment, at different points in time and as grand challenges mutate (Krütli et al., 2010; Stauffacher et al., 2008).

Enabling distributed agency through research projects thus involves concerted efforts from researchers to move from punctuated to continuous involvement. It accommodates differences in interests, time dimensions, logics, and communication of participants (Bartunek and Rynes, 2014; Sharma and Bansal, 2020). For researchers, this means that projects need to foster spaces in which “research with” and “research on” participants is possible (Kistruck and Slade Shantz, 2022). “Research with” participants involve the collaborative inquiry between the research team and non-academic participants in co-developing and co-constructing solution options. “Research on” brings attention to processes not directed by researchers and acknowledges that they do not have the sole responsibility to advance a project. Accordingly, generativity highlights the need for research projects that allow various audiences to construct, through flexible and autonomous processes, new viewpoints and initiatives able to match the complexities of problem situations (Bradbury and Divecha, 2020; Fazey et al., 2020; Goldstein and Butler, 2010). Interactions that promote unpredictability and emergence make distributed agency crucial for generative research.

Implementing this tenet means researchers cultivate care for others, independent of their “closeness” to the project, and build trustful relationships inclusive and respectful of varying timelines, objectives, or capacities. Distributing agency inherently means sharing ownership and power over the research process. Hence, in instances where power is not ceded, participants in generative research projects must pursue paths for distributing power and provide opportunities for others to express themselves in decision-making processes.

### Tenet 3 on realizing interventions: conduct experiments

Generativity offers a fluid approach to research and treats problems and solutions as provisional and evolving. It prioritizes the realization of opportunities that are possible (instead of desirable). This tenet calls upon generative researchers to utilize experimentation as their modus operandi to explore opportunities for realizing concrete changes within a given context. This shift, with its focus on actionability, in turn requires improvisation, monitoring, and revision in light of changing situations and new information. Improvisation harnesses “the generative forces of incompleteness” (Garud et al., 2008: 356) to create opportunities for continuous improvement and emergent processes. Moreover, because research projects cannot anticipate which solution will be most effective, experimentation has a crucial role in iteratively improving attempts to mediate problems based on continuous feedback (Ansell and Bartenberger, 2016). These experiments take place in the real-world with oversight from researchers and practitioners to evaluate and learn from interventions and make them work (Bergmann et al., 2021).

Although both originate in pragmatist philosophy, there is a subtle distinction between the experimentation in generative research and the experimentation described in robust action strategies (e.g. Ferraro et al., 2015). The goal of experimentation in robust action is to create small wins and thereby build momentum. This is not antithetical to the experimentation we prescribe, yet here we see experimentation in the mold of the scientific enterprise, to create evidence on solutions that work but also understand why they may not (Caniglia et al., 2017). The goal of experimentation as a means of generative research is to find workable solutions to situations that are dynamic, open-ended, and therefore unpredictable. For researchers, even when experiments do not deliver the expected results, or fail, learning takes place. In fact, following Etzion (2018), failures may well be a good measure of effort and ambition.

Experimentation dissolves the misconception that knowledge and action are separate domains and that the initiation of research projects and subsequent change are sequentially linked (West et al., 2019). Instead, experimentation enables researchers to apply and draw insights from within action situations. Societal change is ongoing and research projects are situated within, informed by, and contribute to such processes. This mode of experimentation serves generative researchers collaborating with other actors in two ways: First, it provides the means to intervene and thereby create changes in situations that are being investigated (Caniglia et al., 2017). Second, through mentoring and evaluation, it produces empirical evidence on the effectiveness of potential solutions and the problems they seek to address (Caniglia et al., 2017; Luederitz et al., 2017b). Experimentation thus enables learning to unfold across interventions as researchers seek to create overlaps and reinforce desirable dynamics within and beyond project activities.

Implementing this tenet in research projects requires that researchers leave their comfort zones to work collaboratively when intervening and putting to the test transformative actions. Conducting experiments requires researchers to gain confidence in working in evolving situations as means (i.e. how to experiment) and ends (i.e. what to achieve) are iteratively determined. Researchers, thus, must embrace improvisation in aligning short-term interventions within specific contexts with the goal of achieving prospective impacts, meaning that experimentation—even if unsuccessful—needs to contribute to creating conditions for change.

### Tenet 4 on creating transformative change: pursue prospective impacts

Generativity is ultimately a future-oriented capacity of researchers seeking to create impact. This tenet calls upon generative researchers to create new operating spaces that are conducive to transformative change. In the literature, generativity materializes in efforts intending to care for and empower others. Building on its psychological foundations, generativity reminds us of the human satisfaction inherent in reciprocating and paying forward “good deeds.” Generativity-oriented researchers strive to allow actors to enact this capacity in a prospective fashion, engaging in present action to improve future disparities. It means that researchers must refrain from merely directing others in their efforts to solve problem situations and must themselves become agents in change processes.

Embracing generativity thus encourages researchers to adopt a prospective orientation (Pavez et al., 2021). Usually, impact-driven approaches favor immediate and clearly observable changes over longer term results. The long-term orientation of prospective impact, however, enables researchers to engage with the future “as if the envisioned events have already occurred, and look back from that perspective . . . [to] influence interpretation of the past when the imagined future actually emerges” (Gioia et al., 2002: 630). Indeed, generative researchers need to guide “normative conceptions of the future . . . to create new future visions—strengthened through theory—that open up radically new prospects for human agency to shape the world” (Gümüsay and Reinecke, 2021: 2). This places attention squarely on questions around the conditions that are needed to enable future actions and ripple effects beyond projects themselves.

The focus on prospective impacts makes otherwise abstract change processes tangible and meaningful. Research projects that embed generativity are not conceived as single attempts to resolve a given problem but are theorized as part of unfolding (societal) processes with the objective of creating fundamental change in the future (Sharma et al., 2022). Whereas such change can seem distant, or even utopic, as primary concerns are occupied with possible or feasible futures, the objective of working toward prospective impacts foregrounds desirable transformations of grand challenges (Gümüsay and Reinecke, 2021). Emphasizing desirability enables “people [to] become excited about some vision they truly want to accomplish” and gives them the opportunity to be “part of something larger than themselves, of being connected, of being generative” (Senge, 1990: 13). Therefore, research on grand challenges does not predict or merely anticipate impact but focuses on creating conditions for change to take place. At the same time, generative outputs invite continuous monitoring and evaluation of participants’ development instead of keeping score of the “number of articles we publish or grants we secure” (Ball, 2012: 289). This fosters reflection, increased awareness, and the realization that knowledge without engagement and application is insufficient for change.

Implementing this tenet requires researchers to take current and future generations seriously when contributing to the development of spaces conducive to change. Engaging with the normativity of prospective impacts means addressing questions over who is to benefit from specific circumstances and whose interests are reflected as projects unfold. Pursuing prospective impacts encourages generative researchers to engage in ongoing evaluations, ensuring that opportunities for reciprocity are enacted and conditions for transformative change are created.

## Generativity in action—the PIVOT project

Generativity played a key role in the enactment of PIVOT, a transdisciplinary research project focused on climate action in SMEs in Canada. The project originated from a collaboration between researchers at McGill University and creatives at the digital interactive studio of the National Film Board of Canada (NFB), a government-owned media producer and distributor. We reflect upon the project, and particularly on three specific moments that shaped its direction: genesis, transition, and continuity. We use *italics* to highlight the most explicit manifestations of the tenets throughout the evolution of the project.

### Genesis

In 2018, NFB approached researchers at McGill University in search of a research partner to collaborate on a media project on Canadian society and the climate crisis. The researchers proposed the focus on SMEs for two primary reasons. First, the research team felt that SMEs would be useful to highlight, because they are diverse yet relatable entities and generally looked upon with favor across the political and ideological spectrum. Second, the researchers perceived an opportunity to conduct methodologically novel work on SMEs and climate change, complementary to the majority of work on this topic, which relies on survey data and interviews. Rather quickly, the two groups agreed in principle to work together, albeit formalities in the legal and financial domain took quite some time to iron out.

Initial discussions were slow and at times frustrating but created space for *distributing agency* across the transdisciplinary team in the form of shared project ownership. Over the course of many months, substantial time was devoted toward understanding and appreciating the worldviews and priorities of the research team and the NFB team, assigning roles and responsibilities, designing workflows, and establishing trust. This work was facilitated by regular meetings that took place roughly every 2 weeks. Early on, the project benefited from embracing openness toward exploring different ideas about artistic expressions and research possibilities. The team also grew by involving researchers from different faculties and disciplinary backgrounds and by integrating new perspectives and varied methodological orientations. These *diverse inputs* sedimented in discussions about the focus of the project, centering on climate change communication around SME actions. Team members encouraged each other to consider interventions of various online (such as video, image, text) and hybrid formats. Given NFB’s expertise in media and communication, the group deliberated about the presentation of the research team: should they be the protagonists of the project, akin to documentary films that follow intrepid biologists, oceanographers, and others in the field? Should the researchers be portrayed as sources of wisdom and expertise on transitioning businesses to the green economy? Should the researchers take a backseat, and be only minimally visible, or entirely behind the scenes? While initially no answer to such questions was forthcoming, the intention to *diversify inputs* was strengthened. Accordingly, research insights were seen as one among other equally relevant ways of knowing. Eventually, foregrounding the expertise of SME owners emerged as a preferred objective.

These conversations created opportunities to identify and consolidate what kind of *experiment* the team wanted to realize. Both the NFB and the researchers were aware of the limits of much climate communication. Many communication-based efforts in the climate action space emphasized “how-to” lists or “X easy steps toward reducing your climate footprint.” Other efforts focused on pledges and commitments—typically vague, often insubstantial, usually both—with very little follow through, if at all. The researchers claimed that such efforts did not have much of an effect, the NFB had a similar view. In other words, the partners concurred that typical communication efforts around climate did not lead to substantive *prospective impacts*, and wanted to pursue an effort that would evolve and grow. Accepting the shortcomings of prior efforts was motivating and invigorating because both partners were interested in novelty and impact. The researchers sought to create real-world impact and study it along the way; the NFB sought to manifest its values of artistic creativity, originality, and ambition, and in doing so also create change in Canadian society.

Several team members were concerned that without agreement on an overarching goal, the project would not accomplish anything concrete in a reasonable timeline. Therefore, the partners spent time to devise and hone an initial project goal: “Amplify in real time the actions of small businesses to accelerate tipping points that reveal opportunities in the new climate economy.” Guided by the tenet *diversifying inputs*, this goal was thought to plausibly resonate with large swaths of Canadian society. The intention was twofold, first, not to alienate established actors who emphasize the central role of the economy in society and are comfortable with the status quo. Second, to entice actors seeking more dramatic shifts and transitions as befit the urgency of the climate crisis. With this goal, the NFB saw the project as an opportunity to create new content about Canadians taking climate action from sea to sea to sea, a movement that they would be able to document in order to fulfill their mandate of holding up a mirror to Canadian society and its transformations. The research team viewed the project as an opportunity to study the dynamics of diffusion and social change in real-time, a domain of general theoretical and empirical importance (e.g. Centola, 2018; Cialdini, 2016; Frank, 2021).

After agreeing on the project orientation, the *experiments* began to take shape. The team elected to use first-person narratives to demonstrate, by example, that SME climate action was not as rare and/or difficult as it might seem, and thereby to encourage other business owners to join efforts in addressing climate change. To promote *diversity of inputs* and highlight *distributed agency* for climate action, emphasis was placed on portraying diverse SMEs across different industries, business sizes, genders and ethnicities, geographical locations, and value systems in a non-judgmental, accessible format. In line with the NFB’s exacting professional standards, stories needed to make use of high-quality photographic images and text, and to be easily accessible on computers, tablets, and mobile phones, with the expectation that content would be consumed over a span of seconds or minutes. These requirements led to the concept of a collection of short stories of individual entrepreneurs across the country taking climate action and explaining why they did so.^1^

**Figure 1.**
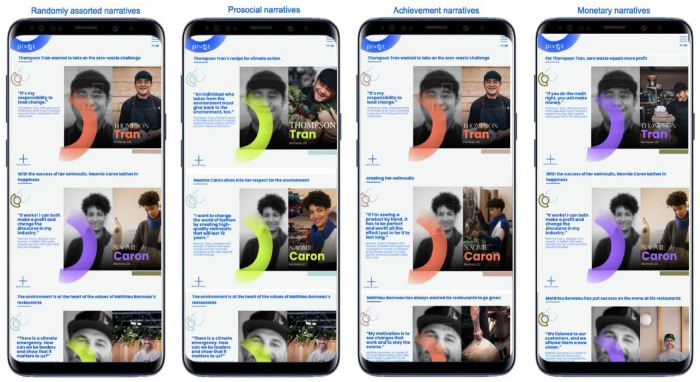
First version of the PIVOT website as presented on a mobile device. The website design included four different versions displaying (left to right) randomly assorted narratives, prosocial narratives, achievement narratives, and monetary narratives.

To probe the viability of the idea, researchers, students, and creatives interviewed 89 SME owners identified through personal contacts, networks, and web searches. After reviewing the results of these interviews, 13 SME owners were selected to be the primary protagonists on the project website. The selection was informed by considerations of geography, sector, gender, and ethnicity, and based on NFB’s assessment of how telegenic and relatable they were. Subsequently, a website (www.gopivot.org) was designed, coded, and tested, as a way to conduct an *experiment* and intervene in how SME climate actions are framed and communicated (Figure 1). The team perceived the website to be a unique opportunity to conduct research on motivation driving climate actions.

To populate the website with content, an interview protocol was developed to elicit the climate stories of the 13 selected SMEs. As team members met to discuss the interview questions, one issue came up again and again, and was never adequately resolved. NFB were interested in uncovering and highlighting the economic rationale for taking climate action, assuming that this type of content would be particularly effective at swaying other business owners. But examination of the initial interviews with 89 SMEs, as well as a survey of academic literature, suggested that this was not the only motivation to highlight. No agreement was found over which motivation to prioritize, until the team agreed to transform this debate from an argument into a research question, to be resolved through a rigorous scientific study. Consequently, the interview protocol for the 13 SMEs asked the owners to reflect on their motivations from a variety of viewpoints, as a way to make visible *diverse inputs*.

Upon analysis, the researchers identified three distinct reasons for SME-driven climate actions: prosocial motivations (i.e. altruistic and biosphere values), achievement reasons (i.e. self-enhancing values), and monetary rationales (i.e. economic values). Inspired by this observation, the NFB created three distinct stories for each SME owner that highlighted just one of the motivations, as opposed to aggregating them into one narrative. These stories, in turn, were used in a quantitative empirical study that examined econometrically which of the motivations was most compelling for audiences. To conduct this research, dedicated marketing campaigns were designed on different social media platforms (Facebook, LinkedIn, Twitter) to randomly display just one of the three narratives to different businesspersons across Canada, and to assess the degree of engagement with that specific narrative (Luederitz et al., 2023). Because it emerged from a close collaboration with experts in narrative communication, this research provided insights into how best to craft climate stories to stimulate action among SMEs in Canada and elsewhere, *a prospective impact*. From an academic perspective, it revealed the plurality of logics (Kraatz and Block, 2008) present within persons typically perceived as economic agents.

### Transition

After several months, the need for a second phase of *experimentation* became apparent. The first iteration of the PIVOT website, and the start of the study on climate narratives, took place in mid-2021, accompanied by a marketing and press campaign, championed by the university and NFB. As the project team continued to publish individual stories, compiling in the end over 90 SME portraits, combined with the insights from the study on narratives, the team recognized that proceeding along this path offered diminishing returns for visitors to the website; there were only so many stories that a typical person would want to read. Similarly, the ongoing evaluation suggested it was expensive and time-consuming to update the stories in real-time in a way that would be compelling. The first *experiment* had run its course.

This juncture suggested a return “to the drawing board.” The ongoing evaluation made it evident that the team needed to revisit the “theory of change” connecting its activities and the *prospective impacts* it was pursuing. These reflections helped the team to recognize some clear path dependencies and sunk costs: we were largely committed to a digital project, we had already defined ourselves as a web presence, we had a core user base, and we wanted to increase the impact of our *experiment* on climate communication.

Combining these bits and pieces of assets and intentions led, through collective deliberation, to the logical conclusion that the next *prospective impact* of the project needed to support, sustain, and empower its users, that is, to hark even closer to the tenet of *distributed agency*. In other words, it needed to evolve from a website that a user surfed passively, to some form of social media platform. At first glance, it appears that this conclusion leads to a narrower and less generative path. To some extent, any decision does, of course. Yet, the team realized that this vision could enable and sustain participation from diverse actors and create opportunities for new paths to be explored. In fact, in terms of vocabulary, the team started using the word “platform” more than website, with all the affordances that the word can provide. Once a platform is available, many things can be presented upon it, moved, shuffled, and replaced as needed, with entry points for a (diverse) variety of actors (cf. Porter et al., 2020).

**Figure 2.**
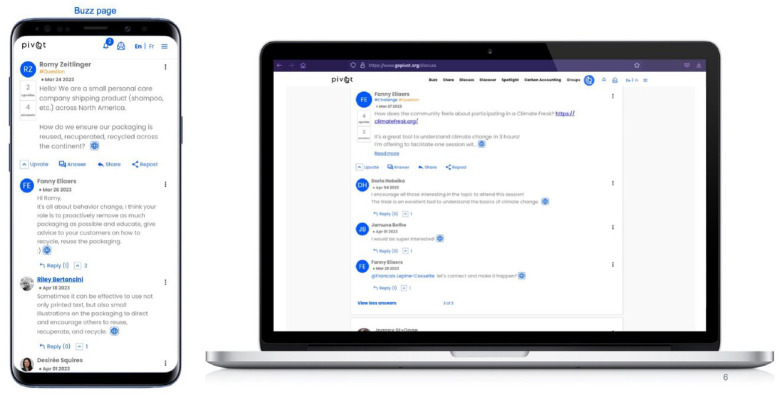
The second version of the PIVOT website as viewed on a mobile device (left) and laptop (right). The different functions of the platform include a buzz page (with popular posts), a share page (for chronicling users’ stories), a discuss page (to debate questions), a carbon calculator tool (for estimating emissions), and a groups page (for subject or organization specific dialogue).

For the next *experiment*, the team designed the PIVOT platform to enable users to conveniently interact with each other on topics related to climate and small business (Figure 2). The platform was developed by professional and student coders and loosely modeled around https://www.patientslikeme.com, a website that empowers people to “navigate their health journeys together through peer support.” From a practical “what’s in it for me,” or “use-case” standpoint, the platform *experiments* with multiple functions: it can enable users to build their network across the country, share information, follow the climate actions of others, engage with a variety of content and emotions, and become climate-positive actors themselves. Typically, in other social media sites with a business orientation, individuals present themselves and their actions—almost always successes—in a positive light. In PIVOT, we sought to foster the emergence of diverse modes of communication, by encouraging dialogue of all emotional valences around actions, challenges, and questions. In this regard, the PIVOT project took a huge leap of faith in terms of *distributed agency*, hoping that users would invest time and energy in vibrant, ongoing engagement and content creation, and would be willing to present—truthfully and transparently—as occasionally confused, frustrated, overwhelmed, or unsuccessful in certain efforts.

Despite the research project’s focus on climate change, the platform *diversifies inputs* by allowing all aspects of sustainability to be brought to light and discussed. Content moderation is done with a very light touch, as opposed to trying to constrain the topic to climate action narrowly defined. Our observation of the posts reveals that, in reality, most users do not distinguish between climate concerns and other sustainability concerns such as recycling and waste disposal. Indeed, it seems that much discourse revolves around this latter topic, even though social justice issues, transportation, living wage, procurement, mental health, weatherization, and other topics are no less important. In fact, the climate discourse on PIVOT is rather impoverished, focusing on very few topics, almost entirely related to energy and material efficiency within the boundary of the organization. A future research project—surfaced by the notion of *diversifying inputs* embedded in PIVOT—will study the topic of unprompted business discourse around climate change, and the extent to which it conforms, or not, with how the scientific community perceives climate action.

**Figure 3.**
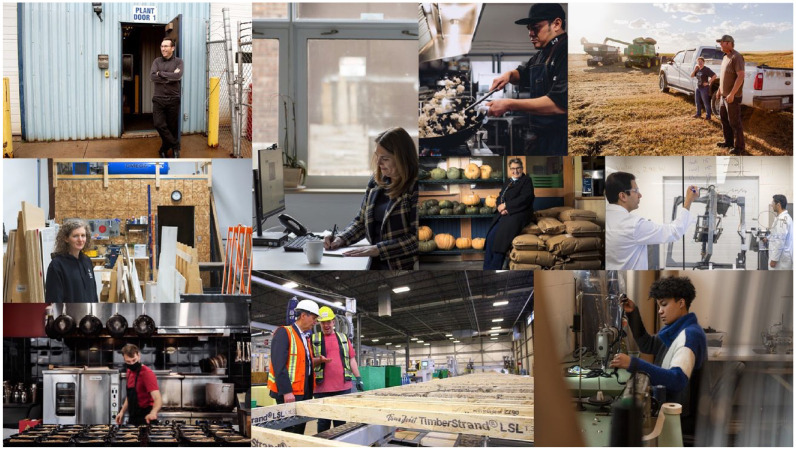
A selection of SME-owner portraits.

As do all digital platforms, PIVOT *distributes a great deal of agency* to users. Most central are the SME founders and leaders themselves, who embody *diversity* in size, organizational procedures, industries, and goal orientation. From its beginnings, the project team discussed the importance of reaching “unusual suspects” in the climate space, such as gyms, pharmacies, and metal fabricators, as opposed to focusing on climate-tech, food, and cosmetic companies, which often seem to receive much greater attention in both the media and in management research on climate action (Figure 3). This objective was attained, more so than another of the project’s objectives, which was to be pan-Canadian. In reality, the project has had much greater uptake and engagement in the city of Montreal, and in Quebec, where it is headquartered, than it has in more distant provinces in the country.

PIVOT has also actively sought out additional *diverse inputs* from other actors. Two partners—one pan-Canadian and one Quebec-oriented—that joined the project in 2020 focus on the role of small businesses in decarbonizing the economy. These partners saw PIVOT as closely aligned with their own goals, as have several other sustainability-oriented regional business networks and industry associations. They brought their own networks to the platform. Less successful have been attempts to engage with partners that are not primarily oriented toward sustainability, such as chambers of commerce. Likewise, dozens of students at McGill enthusiastically joined the project taking on programming, graphic design, and especially user support and social media roles, domains in which they have greater competencies than the academics, and therefore are asked to assume nearly full autonomy and *agency*.^2^

Alongside these efforts, the team also began discussing opportunities to interweave online activities more strongly with climate action in local contexts. The researchers and creatives agreed on expanding PIVOT into Biosphere Reserves—UNESCO designated model regions that reconcile economic development with environmental protection. The goal was to explore opportunities for identifying and supporting collective actions among SMEs, not-for-profits, and public organizations acting on climate change. For the initial outreach, the team sought to reach out to businesses that were not aligned with climate action in order to further *diversify inputs*. This objective was achieved by systematically inviting over 400 SMEs located in the Mont-Saint-Hilaire Biosphere Reserve to participate in in-depth interviews which also helped to establish good rapport with local businesses. While the connection with the PIVOT platform was not immediately apparent and potentially even seen as contradicting as it diverted attention away from the main (online) focus of the project, the team broadly supported this initiative as it posed an opportunity to *conduct experiments* within the unsustainable realities of the Mont-Saint-Hilaire Biosphere Reserve.

### Continuity

As of summer 2023, the online platform remains the core *experiment* of the PIVOT project. However, its growth has slowed significantly, and a particularly acute problem is the slowdown in content posted by users. This dynamic opened possibilities for the research team to explore new research trajectories—strengthening the focus on particular locations and networks—and ensuring the long-term viability of the platform by situating its ownership within a cross-faculty partnership. At the same time, we continued to encourage more user content, including in-person events, prompts through email, and a weekly newsletter with select questions from other users. These initiatives are ongoing.

As noted earlier, a benefit of the platform is the affordability that it provides in *conducting side and follow-on experiments*. Therefore, the PIVOT team generally enables any research project that desires to piggyback on the platform. One researcher wanted to integrate a carbon calculator and study its uptake and effects. A student developed a calculator suitable for the province of Quebec and its energy sources, other students wrote the code, designed the user interface, and tested it, and yet other students demonstrated it in workshops. All this feeds back to data collected on the platform, enabling the study of usage patterns, and subsequent behavior change when users reassess their carbon footprint at a later stage, providing a pathway to a meaningful academic publication. Similarly, NFB is planning a follow-up project in the Mont-Saint-Hilaire Biosphere Reserve, to tally, extrapolate and visualize SME carbon emissions.

Meanwhile, the research in the Mont-Saint-Hilaire Biosphere Reserve continued and moved to the next phase. Based on the *diverse inputs* from participating SMEs, the research team identified a low level of perceived agency among businesses which may explain the lack of climate initiatives in the region. The gathered insights informed *experimentation* with different engagement formats, including—aside from interviews—online and in-person workshops. The focus of these interventions was to *distribute agency* among private and public actors and create opportunities to discuss how such organizations could establish conditions conducive to climate actions in the region, that is, *prospective impacts*. This effort led the research team to examine the potential of different forms of engagement in supporting transformative learning among participants (Taimur et al., under review). Encouraged by the overwhelmingly positive response from the participants of the first two workshops, the research team is now exploring opportunities to use the platform with other Biosphere Reserves in Canada to strengthen their engagement with SMEs. In Mont-Saint-Hilaire Biosphere Reserve, the research team collaborates with a tourism business, led by an undergraduate student as liaison, and started to design *experiments* to engage their clients more effectively in their sustainability efforts. These activities are ongoing.

Of course, prioritization and allocation of effort around a project with offshoots can create tension. With different team members hoping to pursue distinct threads, some are inevitably slower to reach fruition. As they occur, some postponements are accepted with equanimity; others create unease regarding the pace and quantity of academic outputs, due in no small part to the research team encompassing members across different faculties. These situations could, when approached constructively, lead to new ways of collaborating among team members or potential spin-offs to third parties oriented toward small businesses and/or climate action, and who might be well suited to continue developing and maintaining *experiments* that align with their own missions.

Perhaps the riskiest and biggest *experiment* continues to be the online platform itself, and our effort to create a viable, vibrant social network. We perceive this as a high-risk, high-reward endeavor. It requires patience and creates some anxiety, especially for younger scholars, and those habituated to speedy and frequent academic outputs, not to mention the project’s funders. Yet, if we are able to create a network over which we have full technological control, the possibilities for research on online behavior would be endless: running A/B tests, analyzing discourse and vocabulary, conducting longitudinal network analyses, examining contagion processes, and so forth. As the affordances of users become apparent, research and *real-world impacts* would—naturally and in fact unavoidably—become increasingly intertwined. To support these goals, the research team transitioned the management of the PIVOT platform out of the research project and embedded it in a cross-faculty partnership mandated to support businesses in developing socially viable and environmentally integrated opportunities.

## Discussion

In this section, we reflect on the four tenets, their interdependence and how they informed our research practice in realizing PIVOT as a generative project. Next, we discuss the broader implications of our work for impact-driven research by situating generative research within existing approaches aiming to address grand challenges.

### Reflections on the tenets of generative research

Generative research is contingent on the realization of all four tenets. The four tenets—*diversify inputs, distribute agency, conduct experiments, pursue prospective impacts*—consider different aspects of research projects and reinforce each other. As demonstrated through the PIVOT case study, the tenets are not chronologically ordered, and their enactment does not align with the linear progression of a project. Instead, in each project phase, all tenets are relevant. For example, pursuing prospective impacts is not realized at the end of a project cycle but generative researchers consider it and seek its realization throughout. Similarly, diversifying inputs does not constitute a founding project activity, but the tenet serves as a constant reminder and aspiration for researchers, to be pursued by integrating pluralistic understandings, welcoming different kinds of knowing, and questioning values, among others. Wholehearted generative research means pursuing all tenets simultaneously because each serves a different, complementary purpose.

The tenets as illustrated through the PIVOT project systematically support and reinforce impact-driven researchers’ commitment to generativity. Yet, the tenets are not always equal in scope and contribution and vary throughout the course of a project. To elaborate this point, we return briefly to the *pursued prospective impacts* of PIVOT and explain how the other tenets were entangled in and contributed to their achievement. The overarching *pursued prospective impacts* of PIVOT focused on empowering SMEs since they are often spoken for and about but rarely given a voice on issues of climate change. On multiple occasions, the project team expanded on this commitment such as when (1) when creating spaces for voices otherwise quietened, (2) when seeking to engage “unusual suspects” to reach beyond those dominating climate discourses, and (3) when developing the platform to shift content creation from professional content creators to SMEs themselves. While in themselves these actions may not be transformative, they carry potential for such change through reinforcing and ripple effects.

To demonstrate how PIVOT created spaces in which alternative ways of organizing can flourish in the future, we briefly elaborate on each of these prospective impacts and their entanglement with the other tenets.

(1) PIVOT sought to elevate the voices of SMEs in climate change discourses. This motivation emerged from *diverse inputs*, including the research team itself, and insights formed by extended engagement with SMEs. When the research team selected SMEs across geographies, sectors, genders, and ethnicities as protagonists for *experiments* around climate communication, PIVOT *distributed agency* over what stories were told and created a space in which such conversations could unfold. This work collectively resulted in research outputs on climate communication (Luederitz et al., 2023).
(2) PIVOT sought to expand the scope of engagement with SMEs beyond those typically envisioned as climate change leaders. This subsequent *prospective impact* evolved as the project developed and the research team was exposed to *diverse inputs*, be it more mainstream communication on sustainability and business, or the teams’ discussion about opportunities to provide a platform for everyone. This *prospective impact* also informed the research in the Mont-Saint-Biosphere Reserve which sought to *diversify inputs* by systematically involving diverse SMEs across the studied region. Here, *experimentation* focused on engaging SMEs in building awareness of the need for fundamental change, given the focus on “unusual suspects” meant the target audience rarely viewed themselves as endowed with agency to address climate change (Gauthier et al., 2022; Taimur et al., under review). On the website, the team sought to build toward this goal by enlarging the climate communication *experiment* and *distributing agency* for the creation of relevant content by empowering graduate and undergraduate students to identify and portray new SME protagonists.
(3) Ultimately, PIVOT embraced the decision to shift content creation from professionals to SMEs themselves to continue working toward the *prospective impact* of SME empowerment. *Diverse inputs* enabled this transition, including insights that emerged from other aspects of PIVOT and efforts to welcome students’ contributions to the development of the design and platform features. Doubling down on this commitment meant fully *distributing agency* of content creation and opening the platform for SMEs to take charge. This in itself constitutes PIVOT’s most ambitious *experiment* which continues to evolve as new partners join the project and new research will be developed around specific platform features.

These three examples reveal how *diversifying inputs, distributing agency, conducting experiments, pursuing prospective impacts* interrelate, and demonstrate how generativity can play out in impact-driven research projects. They also demonstrate that pursuing prospective impact does not interfere with conducting rigorous research, and that a research agenda need not impede real-world change.

### Generative research in the context of impact-driven approaches

PIVOT is an instantiation of engaged scholarship that seeks impact. It is aligned with engaged scholarship and its intimate engagement with crucial societal concerns, while being dialogic, reflexive, relational, and participatory (Cunliffe and Pavlovich, 2022). Indeed, in PIVOT, we engineered an opportunity for SME owners to realize, without being lectured at—shown, not told—that they are an engaged community, committed to climate action. Whether and how this manifests, what relations they will build, and how they will act together are as much a source of novelty, theorizing and knowledge production for them as it is for the academics involved in the project.

In addition to the affinity with engaged scholarship, we also see similarities between generative research and intellectual activism as espoused by Contu (2020). Although explicitly political—with a bias toward direct engagement with issues of social justice—a key plank in her approach is performativity, rejecting the “representationalist view of knowledge that is based on the separation between subject/object and the neutrality of knowledge” (Contu, 2020: 2). Citing Barad (2007), Contu argues that performative knowledge production participates in bringing into being and making intelligible specific realities, relations, and subject-objects. Research is inextricably linked with the deeds a researcher does. Moreover, this work must be concrete and practice-based. In Contu’s (2020) own powerful words, intellectual activism

. . . is not an ego-propping exercise characterized by finger pointing and taking an empty moral high ground. There is no purity. This is about humility. The humility of not knowing; of not having all the answers; of making mistakes; of facing the Other openly; of being challenged and disturbed; of listening and learning from experiences, knowledge, realities and needs that move us literally to another “place” we build together. (p. 12)

This view is echoed more recently by Gray (2023): “Working collaboratively with other stakeholders, rather than positioning ourselves as ‘experts,’ will require us to bring inquisitive, ‘don’t know mind’ to the issues we tackle coupled with a healthy dose of humility” (p. 180).

In our view, generative research is thus ardently democratic. Indeed, in PIVOT, we did not treat the involved actors as our audiences or ourselves as experts but viewed them as peers, able – and hopefully willing – to assess the outcomes and effectiveness and reach conclusions themselves. While we analyzed whether introspection and analysis occur as a result of some of our experiments (Taimur et al., under review), we cannot be certain that it does on all occasions.

### Generativity as opportunity and obligation

Grand challenges create new opportunities, or, perhaps more aptly, demands and obligations, for us as management scholars to make significant contributions to societal transformations toward greater sustainability. To fulfill this obligation, we drew on generativity to provide guidance to researchers on how to gather diverse inputs and empower a broad variety of actors to jointly conduct concrete interventions that constitute the building blocks of transformative change. The tenets we set forth do not constitute a new research approach with its own epistemology and methodological procedures. We simply encourage researchers to incorporate generativity into new and ongoing projects. Generative research defies our current way of conducting projects and has the potential to guide scholars wishing to have impact in the context of grand challenges.

Perhaps the greatest value of generativity is that it allows us to move beyond being spectators and cheerleaders for change. Researchers can insert themselves into the arena and actively support the conditions that enable transformation. Doing so can more clearly reveal how the people entangled in grand challenges (including the researchers themselves) think and act, and the consequences that ensue. Researchers are privileged insiders yet also complicit in the system that needs to change. Generativity moves beyond the “do as I say” approach of recommendation for practices and provides the foundation for an optimistic stance toward people’s agency in addressing grand challenges.

## Notes

1. One source of inspiration was the website https://www.humansofnewyork.com/.

2. At the same time, rapid turnover among students due to graduation is a challenge.
